# NePCM Based on Silver Dispersions in Poly(Ethylene Glycol) as a Stable Solution for Thermal Storage

**DOI:** 10.3390/nano10010019

**Published:** 2019-12-19

**Authors:** Marco A. Marcos, David Cabaleiro, Samah Hamze, Laura Fedele, Sergio Bobbo, Patrice Estellé, Luis Lugo

**Affiliations:** 1 Departamento de Física Aplicada, Universidade de Vigo, E–36310 Vigo, Spain; mmarcosm@uvigo.es (M.A.M.); luis.lugo@uvigo.es (L.L.); 2 Institute of Construction Technologies, National Research Council, I–35127 Padova, Italy; laura.fedele@itc.cnr.it (L.F.); bobbo@itc.cnr.it (S.B.); 3 Université Rennes 1, LGCGM, EA3913, F–35704 Rennes, France; samah.hamze@univ-rennes1.fr (S.H.); patrice.estelle@univ-rennes1.fr (P.E.)

**Keywords:** silver nanoparticles, PEG400, NePCM, heat storage, thermal conductivity, dynamic viscosity, surface tension

## Abstract

The main objective of this study is to design and characterize silver suspensions based on poly(ethylene glycol) PEG400, Ag/PEG400, as energy storage media for low-temperature applications. A polyvinylpyrrolidone (PVP) treatment was applied to ~22 nm silver nanoparticles to ensure good stability in poly(ethylene glycol). An array of different experimental techniques was utilized to analyze the molecular mass and purity of base poly(ethylene glycol), morphology of dry PVP-capped Ag nanoparticles, hydrodynamic average size of dispersed Ag particles, as well as thermal stability of PEG400 and Ag/PEG400 dispersions. Samples exhibited good temporal stabilities with average hydrodynamic diameter around 50 nm according to dynamic light scattering analyses. Melting and solidification transitions were investigated in terms of temperature and enthalpy from differential scanning calorimeter (DSC) thermograms. The thermophysical characterization was completed with thermal conductivity (*k*), dynamic viscosity (*η*), isobaric heat capacity (*C_p_*), density (*ρ*), and surface tension (*σ*) measurements of designed materials using a Hot Disk thermal conductivimeter, a rotational rheometer, a DSC calorimeter working with a quasi-isothermal modulated method, a U-tube densimeter and a drop shape analyzer, respectively. For a nanoparticle loading of only 1.1% in mass, sub-cooling reduced by 7.1% and thermal conductive improved by 3.9%, with almost no penalization in dynamic viscosity (less than 5.4% of increase). Maximum modifications in *C_p_*, *ρ,* and *σ* were 0.9%, 2.2%, and 2.2%, respectively. Experimental results were compared with the values provided by using different theoretical or semi-empirical equations. In particular, good descriptions of dynamic viscosity as functions of temperature and nanoparticle volume concentration were obtained by using the Vogel–Fulcher–Tammann equation and a first-order polynomial *η*($\phi_{v,np}$) correlation, with absolute average deviations of 2.2% and 0.55%, respectively.

## 1. Introduction

Better integration of renewable energy in power systems and enhancement of energy efficiency in thermal facilities are essential pathways to improve energy-related environmental issues [1]. In this sense, thermal energy storage (TES) is a useful strategy to address the intermittency of renewable sources and assist an effective utilization of energy by relieving the mismatch between power supply and demand. TES methods are commonly categorized as latent heat using phase change materials (PCMs), sensible heat, and thermochemical storage technologies [2]. PCMs or latent heat storage media are attracting particular attention due to the high energy storage density (5 to 14 times larger than with only sensible heat [3]) and fewer degradation/reversibility issues throughout a large number of cycles when compared to thermochemical approaches. In recent years numerous materials have been proposed as potential solid–liquid or solid–solid PCMs for applications such as smart housing [2], heat management of electronics [4], or energy generation [5], among others. Refrigeration, one of the major energy consuming processes, has not been an exception. Thus, the use of phase change materials for cold thermal energy storage is also raising increasing interest [6,7,8,9].

PCMs are categorized into different groups according to material nature [10]. Among non-paraffinic organic PCMs, poly(ethylene glycol), PEGs, are some of the most promising candidates with melting transitions that can be selected within a wide range of temperatures, from 277 to 343 K, by means of molecular mass [11,12,13]. In particular, the poly(ethylene glycol) with an average molecular mass of 400 g·mol^−1^, PEG400, used as based material in this work, has its melting transition at ~277 K, which is attractive for cold thermal energy storage [8,14]. PEGs exhibit good chemical stabilities, small volume variations, and high latent heats of fusion [15]. However, as with other organic PCMs, the main disadvantage of poly(ethylene glycol) is their low thermal conductivity, which can unacceptably slow the heat transfer rate of stored energy, precluding practical implementation [14].

Different techniques have been proposed to face the low thermal conductivity of PCMs, such as inclusion of high-conductive particles, encapsulation, shape stabilization, metal foams, or embedding in finned/porous structures, among others [16,17,18]. In recent years, the addition of nanostructures has been found particularly effective not only to increase thermal conductivity, but also to reduce the large sub-cooling characteristic of different PCMs [19]. Latent media obtained from the dispersion of nano-sized particles in phase change materials are known as nano-enhanced phase change materials (NePCMs) or nano-PCMs [4,20,21].

A proper evaluation of NePCMs or other nanotechnology-derived PCMs as both heat transfer and storage media relies on the characterization of melting and solidification transitions, but also on the study of other thermophysical properties such as thermal conductivity (*k*), viscosity (*η*), isobaric heat capacity (*C_p_*), density (*ρ*), or surface tension (*σ*).

Thus, modifications in rheological behavior or dynamic viscosity can considerably affect pumping power and even flow nature of designed fluids [22,23,24] while mass flow rate depends on *C_p_* and *ρ* [25,26]. Although less studied, surface tension also plays an important role in heat and mass transfer processes with low Bond dimensionless numbers, such as microfluidic or systems working under microgravity conditions, for instance [27,28]. A revision of previous studies on storage materials or nanofluids formulated using poly(ethylene glycol) and/or Ag nanoparticles is presented below.

Singh et al. [29] evaluated different techniques to enhance the heat transfer performance of PEG1000, including the addition of carbon powder or the inclusion of either aluminum or carbon fins to the PCM system. An improvement in thermal conductivity of ~31% was observed when 2.5 wt% of carbon powder was dispersed in PEG1000. This enhancement is marginal when compared with the rise in *k* of more than 40 and 30 times obtained with aluminum and carbon fins, respectively. However, aluminum fin stack occupies ~22.7% of storage system volume (which represents ~42.5% in mass), while carbon framework corresponds to ~24.7% of volume (~34% in mass). Thus, larger reductions in storage capacity are expected for the strategies aiming at enhancing the heat transfer rate by using fins (in comparison with approaches based on carbon powder loading). Marcos et al. [30] formulated dispersions of functionalized graphene nanoplatelets (GnPs) in PEG400 and experimentally investigated the influence that GnP loading has on solid–liquid phase change transition temperatures, latent heat of fusion, thermal conductivity, or thermal diffusivity. A maximum thermal conductivity enhancement of 23% and a reduction in crystallization temperature of 4 K was obtained for a graphene nanoplatelet concentration of 0.5 wt%. Yang et al. [31] prepared PEG1000-based PCMs for efficient light-to-heat conversion, collection, and storage using graphene nanoplatelets (GnPs) and boron nitride (BN). The PCM-composite formulated at a BN:PEG:GnP ratio of 30%:69%:1% showed enhancements in thermal conductivity up to 336% and reductions in sub-cooling up to 2 K, in both cases in comparison to pure PEG1000. Multi-walled carbon nanotube/PEG400 dispersions were proposed by Marcos et al. [32] from a chemical, physical, and thermal approach. For the maximum nanoadditive content (1% in mass), thermal conductivity and diffusivity improved by 12.7% and 13.5%, while maximum modifications in density and isobaric heat capacity did not exceed 0.42% and 3%, respectively.

Conversely, authors observed that the incorporation of additives reduced latent heat capacity by ~30%. Babapoor, Karimi and Khorram [15] produced NePCM nanofibers using PEG1000, polyamid6 (PA6), and several nanoparticles (SiO_2_, Al_2_O_3_, Fe_2_O_3_, and ZnO). The highest enhancement in thermal conductivity (above 40% when compared with the former PEG1000) was observed for the PEG:PA6 mixture at a 1:2 ratio and containing 4 wt% of Al_2_O_3_ nanoparticles. Anghel et al. [33] formulated spherical macrocapsules of PEG6000 in epoxy resin using an aluminum nanopowder as filler to reduce charging and discharging processes. Several sets of PEG/SiO_2_ composite form stable phase change materials doped (or not) with other nanoadditives were designed by [34,35,36], Feng et al. [37], Yang et al. [38], or Li et al. [39], among others. PEG/SiO_2_ composite materials containing multi-walled carbon nanotubes [40], active carbon [37], or carbon fibers [41] did not only prove excellent shape-stability and high thermal conductivity but also unique characteristics such as wider absorption range for sunlight, high light-to-heat conversion or energy storage efficiencies.

Despite some carbon nanostructures such as carbon nanotubes or graphene exhibiting thermal conductivities about one order of magnitude higher than those of copper, gold, or silver, large enhancements in the thermal conductivity of common heat transfer fluids were also obtained when dispersing small amounts of metallic nanoparticles [42,43,44]. Zeng et al. [45] investigated Ag dispersions in 1–Tetradecanol as organic phase change material. These authors indicated that thermal conductivity rises with increasing Ag loading but did not report any value of how much those enhancements were. Deng et al. [46] prepared advanced PCMs based on PEG4000 using expanded vermiculite (EVM) as a shape stabilizer and silver nanowires as a thermal conductivity nano-enhancer. Prepared composites exhibited reductions in super-cooling by 7 K (for the EVM:PEG:Ag composition of 28.2%:64.7%:7.1%) and a thermal conductivity 11.3 times higher than that of PEG4000 (in the case of the EVM:PEG:Ag composition of 1.9%:58.8%:19.3%). Qian et al. [47] modified PEG4000 using Ag nanoparticle-decorated diatomite. The presence of additives (either silver or diatomite) did not significantly reduce the sub-cooling (less than 4 K). However, thermal conductivity increased by 127% (in comparison to the mixture of PEG and diatomite used as based) when the shape-stabilized PCM was doped with a silver loading of 7.2 wt%. As a consequence, absorption and release of thermal energy during the phase change was considerably reduced.

The surface tension of silver dispersions in water was studied by Chen et al. [48] and Godson et al. [49]. Chen et al. [48] observed decreases in *σ* with either surfactant addition or Ag concentration (especially when nanoparticle loading overcame 0.2 wt%). Also a decreasing trend with silver content was detected by Godson et al. [49]. In this last study, maximum reductions in surface tension reached 10.3% at 323.15 K for the highest analyzed silver concentration (1.2 vol%). Silver-particle colloids have been considered as potential conductive inks for inkjet printing. Thus, Lee et al. [50] designed silver colloids based on a diethylene glycol–water mixture (50:50 in mass) and stabilized with a 40·10^3^ molecular weight PVP (with an Ag:PVP ratio of 1:8), and the authors studied the effect of Ag concentration on the dynamic viscosity and surface tension of designed samples. Both properties were found to increase with nanoparticle loading, reaching enhancements of ~3% (*σ*) and ~400% (*η*) at the highest Ag concentration (35 wt%). Also important increases in dynamic viscosity were reported by Ankireddy et al. [51] when they studied dispersions (up to 66 wt%) of carboxylic-acid-encapsulated silver nanoparticles in toluene. However, the authors observed that surface tension decreased with the addition of nanoparticles, with maximum diminutions of ~29% for the highest Ag content. Reductions in this property were attributed to a lessening in the interactions between toluene molecules at the droplet surface.

There is still substantial need for further investigative techniques to improve the thermal conductivity of organic PCMs [29]. Thus, the present study aims to develop and characterize stable phase change materials based on poly(ethylene glycol) PEG400 and containing Ag silver nanoparticles as a new stable solution for thermal storage. The effectiveness of Ag loading reducing sub-cooling effect or improving the thermal conductivity and diffusivity is experimentally investigated. Moreover, the thermophysical characterization is completed with the analysis of the dynamic viscosity, isobaric heat capacity, density, and surface tension of presented phase change materials for a wide range of temperatures.

## 2. Materials and Methods

### 2.1. Materials

A NePCM based on poly(ethylene glycol) PEG400 and containing 1.1 wt% of silver nanoparticles was specifically prepared for this investigation by NANOGAP Sub-NM-Powder S.A. (A Coruña, Spain). PVP-capped Ag nanoparticles (DS0476, also commercialized by NANOGAP Sub-NM-Powder) were subjected to a surfactant treatment with polyvinylpyrrolidone, PVP, (at a fixed PVP:Ag ratio of 0.068) in order to ensure good temporal stability in poly(ethylene glycol). Merck PEG400 (Merck, Sigma–Aldrich Darmstadt, Germany) poly(ethylene glycol) for synthesis was used as based fluid. This same PEG400 was employed to prepare the other two studied nanofluid concentrations (0.10 wt% and 0.50 wt%) by dilution from 1.1 wt% dispersion. In order to ensure homogeneous composition of the samples, dilutions were sonicated for 2 min in a low power ultrasonic bath (Ultrasounds, JP Selecta S.A., Barcelona, Spain). Density values of 10.49 g·cm^−3^ corresponding to crystalline silver [52] and 1.20 g·cm^−3^ for PVP were considered in this investigation. In the case of base PEG400, density was experimentally measured in this work. Therefore, at room temperature studied 0.10%, 0.50%, and 1.1% mass fractions ($\phi_{m,np}$) of silver nanoparticles corresponded to volume fractions ($\phi_{v,np}$) of 0.011%, 0.057%, and 0.13%, respectively. A Mettler AE-200 analytical balance (Mettler Toledo, Greifensee, Switzerland) with an accuracy of 1·10^−5^ g was utilized to weigh reagents and samples.

### 2.2. Nanoparticle and Base Fluid Characterization

**Figure 1:**
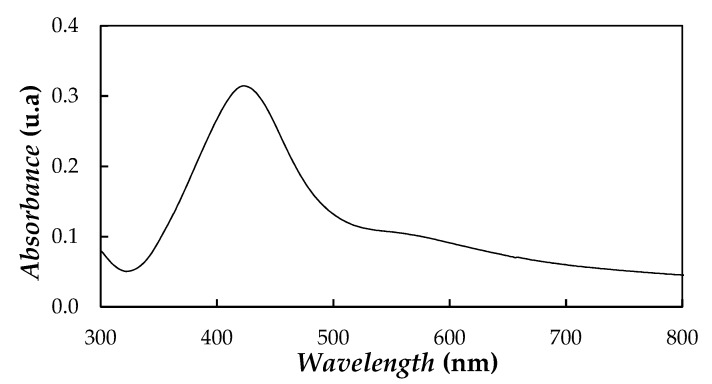
Absorption UV–Vis spectrum of diluted silver dispersion based on PEG400.

UV–Vis spectroscopy is considered a reliable technique in the primary identification of synthesized nanoparticles [53]. In silver NPs, the proximity of conduction and valence bands allows free movement of electrons between both bands. This electron freedom gives rise to a surface plasmon resonance (SPR) absorption band, which confers unique optical properties at certain wavelengths of light [53]. The UV–Vis absorption spectrum of nanoparticles in wavelengths from 300 nm to 800 nm (Figure 1) was recorded on an HP 8452 UV–Vis diode array spectrophotometer (Hewlett Packard, Palo Alto, CA, USA). A highly diluted aliquot of initial Ag/PEG400 dispersion (containing 1.1 wt%) was studied in a standard 10 mm quartz cuvette.

The UV–Vis spectrum of Ag nanoparticles exhibited an absorption peak at ~418–428 nm. The presence of a peak close to ~420 nm and ascribed to SPR was well documented in literature [54,55,56,57] for other silver nanoparticles of sizes ranging from 2 nm to 100 nm.

**Figure 2:**
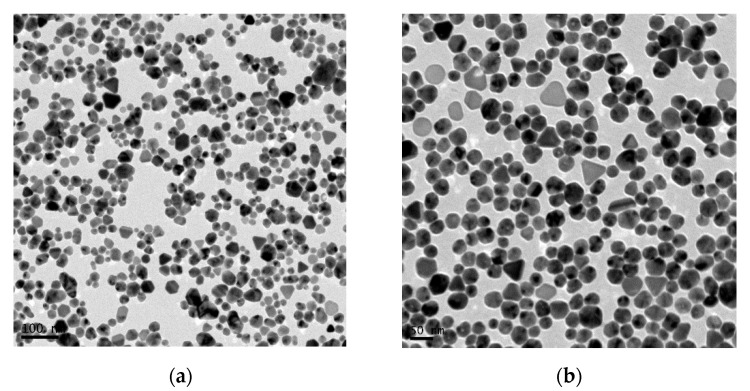
Scanning transmission electron microscope images of DS0476 silver nanoparticles capped with polyvinylpyrrolidone at two different magnifications.

Morphology of silver nanoparticles was examined in a JEOL JEM-1011 (JEOL, Tokyo, Japan) scanning transmission electron microscope (S-TEM) working at an acceleration voltage of 100 kV. One drop of diluted NePCM was deposited on a Formvar-covered 400 mesh copper grid and allowed to evaporate at room temperature. Figure 2 shows a representative S-TEM image in which the quasi-spherical morphology of silver nanoparticles can be observed.

Particle size distribution was obtained by measuring the diameter of a representative number of nanoparticles using ImageJ software (http://rsb.info.nih.gov/ij/). Nanoparticle diameters were mainly in the range of 20 nm to 30 nm (with an average value of 22 ± 7 nm), which agreed with the size of 28 ± 8 nm reported by the supplier for other silver nanoparticles from the DS0476 product.

**Figure 3:**
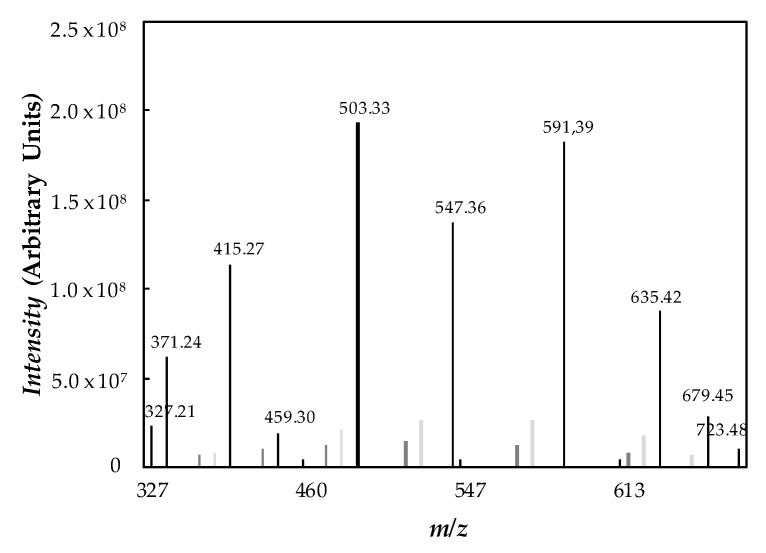
Positive-ion mode electrospray ionization (ESI) spectrum of PEG400.

Molecular mass and purity of poly(ethylene glycol) PEG400 were determined by electrospray ionization mass spectrometry (ESI-MS). Experiments were conducted in a high-resolution APEX Qe FT–ICR mass spectrometer (Bruker Daltonics, Billerica, MA, USA). This device is equipped with a 7 Tesla magnet and configured for external ion accumulation in positive-ion mode. Identification was performed applying a voltage of 300 V to the capillary output. Figure 3 presents the ESI-MS spectrum obtained within the scanning range from 300 to 800 *m*/*z*. Poly(ethylene glycol) is a complex mixture of oligomers, with molecular structure: HO–[CH_2_–CH_2_–O]_n_–H.

All peaks present in the mass spectra correspond to molecules cationized with H^+^ (*m*/*z* = 19.02 + 44.03·*n*, where *n* = 7–16), Na^+^ (*m*/*z* = 41 + 44.03·*n*, where *n* = 8–13), or K^+^ (*m*/*z* = 57.11 + 44.03 *n*, where *n* = 8–14). Therefore, average number molar mass is equal to *M_n_* = 520.50 g·mol^−1^, average mass molar mass being *M_w_* = 532.94 g·mol^−1^ and polydispersity index *M_w_*/*M_n_* = 1.02 (quasi-monodisperse polymer). Molecular mass values obtained by ESI-MS were larger than expected for a poly(ethylene glycol) commercialized as PEG400 [30,32,58,59].

### 2.3. Thermal and Temporal Stability

**Figure 4:**
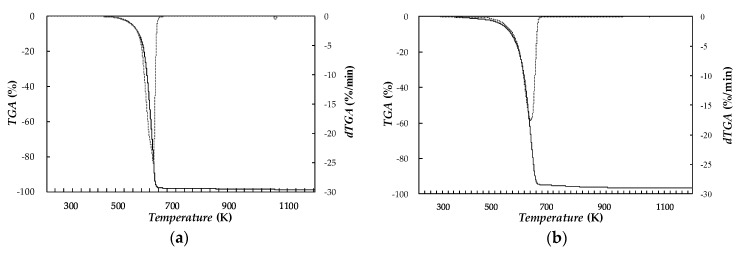
(—) weight loss, TGA, and (- - -) differential weight loss, dTGA, thermograms of (**a**) base fluid PEG400 and (**b**) 1.1 wt% Ag/PEG400 nano-enhanced phase change materials.

Thermal stabilities of neat/base PEG400 and the three Ag/PEG400 dispersions of nanoparticles were investigated by thermogravimetric analysis (TGA) using a Setaram Setsys 1750 TG-DTA (Setaram Instrumentation, Caluire, France). Sample size determinations with this device had a precision of 0.04 µg, while temperature was measured with accuracy better than 2 K. About 30 mg to 35 mg of sample was tarred into ceramic crucibles. Experiments were performed in two steps. Temperature was first raised from 298 to 1023 K with a scanning rate of 1 K·min^−1^ under inert N_2_ atmosphere (flow rate of 30 mL·min^−1^). Then, air atmosphere was introduced in the chamber while temperature was further increased up to 1123 K. Figure 4 shows weight loss and differential weight loss curves obtained for neat PEG400 and the dispersion loaded with 1.1 wt% of silver nanoparticles.

In the TGA thermogram of PEG400, a major weight loss step occurs in the temperature range between 510 and 690 K, while weight loss is lower than 5% for temperatures below 579 K, and *T_onset_* degradation temperature is 609 K. A comparison between PEG400 and Ag(1.1 wt%)/PEG400 curves shows that the addition of Ag nanoparticles only leads to a slight shift (less than 10 K) to the left in the TGA curve.

**Figure 5:**
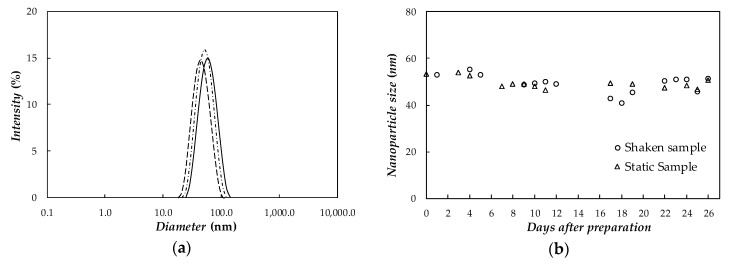
(**a**) Nanoparticle size distribution of static sample based on dynamic light scattering measurements: zero day (—), 7th day (- - -), and 26th day (- · - ·). (**b**) Temporal evolution of average nanoparticle size in Ag(0.01 wt%)/PEG400 dispersion under shaken and static conditions.

The average hydrodynamic size of dispersed Ag nanoparticles in PEG400 was analyzed by means of a Zetasizer Nano ZS (Malvern Instruments, UK) based on dynamic light scattering (DLS). The experimental uncertainty in size measuring suspended particles was estimated to be 2%, further details can be found in Fedele et al. [60] and Colla et al. [61]. In order to ensure appropriate operation conditions, a diluted concentration was selected to carry out DLS investigations. Thus, in this work analyses were performed for an Ag/PEG400 dispersion containing 0.01 wt% of nanoparticles at 298 K and with a scattering angle of 173°. As previously reported for other nanostructured materials [61], hydrodynamic nanoparticle sizes of Ag/PEG400 samples at higher concentrations were expected to be similar to the diameters here obtained for 0.01 wt% loading. Figure 5a shows the nanoparticle size distribution of Ag(0.01 wt%)/PEG400 NePCM. For this last dispersion, the average hydrodynamic diameter is ~50 ± 1 nm. DLS value is almost twice the diameter observed by using transmission electron microscopy. The reason is that DLS size is not based on direct measurements of dry nanoparticles (as in the case of TEM investigations), but on an estimation of hydrodynamic size obtained from an analysis of nanoparticle diffusion behavior. Differences between DLS and TEM size determinations were also reported in the literature for other silver nanofluids by different authors [54,55]. With the objective of evaluating Ag/PEG400 temporal stability, the evolution of average nanoparticle size was monitored in the timeframe of four weeks. Following a procedure similar to the one proposed by Fedele et al. [62], two DLS cuvettes were filled with ~1 mL of Ag(0.01 wt%)/PEG400 dispersion. The first cuvette was kept in static conditions, while the other was hand shaken for some seconds just before performing the measurements. Figure 5a shows the nanoparticle size distributions obtained at three different days after preparation for the static sample, while the temporal evolution of size determinations under static and shaken conditions is plotted in Figure 5b.

As it can be observed in Figure 5, in both samples (under static and shaken conditions) average nanoparticle size remains centered at ~50 nm for the whole analyzed period. This allows us to rule out the presence of any nanoparticle agglomeration or aggregation phenomena and confirm the good stability of designed NePCMs.

### 2.4. Thermophysical Characterization

Solid–liquid phase change characteristics were determined for PEG400 and the three NePCMs by means of a heat-flux differential scanning calorimeter (DSC) Q2000 (TA Instruments, New Castle, DE, USA) equipped with a refrigerated cooling system RSC90. Analyses were conducted at cooling and heating rates ranging from *β* = 1 to 10 K·min^−1^ in a nitrogen atmosphere (mole fraction purity better than 0.99999) flowing at 50 mL·min^−1^. Each measurement condition was repeated at least three times for three different aliquots. Uncertainties in the characterization of thermal events are 0.3 K (temperature) and 1.2 J·g^−1^ (enthalpy). A further description of this instrument and experimental method can be found in Cabaleiro et al. [63].

Thermal conductivity, *k*, was obtained at temperatures ranging from 283.15 to 333.15 K for PEG400 and Ag/PEG400 suspensions using a Hot Disk Thermal Constants Analyzer (Hot Disk AB, Göteborg, Sweden). This device works with the transient plane source (TPS) technique [64]. In this case, a Hot Disk probe consisting of a double spiral made of nickel (2 mm in diameter) and appropriate to measure the thermal conductivity of liquids was selected. Experiments were performed using a low thermal power, 20 to 25 mW, and a short power input time, 4 s. At least four different tests were performed for each sample. The instrument accuracy declared by the supplier was 5%, however, previous tests with deionized water [60] showed deviations with literature [65] better than 2%. More details can be found in Fedele et al. [60].

Shear rate dependence of dynamic viscosity, *η*, was studied for base PEG400 and NePCMs at shear rates between 80 and 1600 s^−1^ and temperatures from 278.15 to 343.15 K. Flow curve rheological tests were developed on an AR-G2 rotational magnetic bearing rheometer (TA Instruments, New Castle, DE, USA). This device is based on a combined motor and transducer instrument and utilizes an induction motor to minimize the friction. Tests were conducted in a cone–plate geometry with a diameter of 40 mm, a 1° steel cone and a truncation gap of 35 μm. Dynamic viscosity results reached repeatability, reproducibility, and comparability requirements of the ASTM D445 standard. An intermediate instrument calibration was performed every three measures to confirm results reliability. After each calibration and series of assays, a check was carried out with distilled water to verify that the rheometer was working in optimal conditions. Water measurements using this experimental device [66] showed accuracy better than 2% with Refprop 9.0 [65].

Isobaric heat capacity, *C_p_*, was analyzed for base PEG400 and silver nanoparticles in the temperature range between 283 and 333 K. Measurements were performed using the DSC Q2000 calorimeter above described, working with a quasi-isothermal temperature-modulated differential scanning calorimetry (TMDSC) method. In this investigation, TMDSC analyses were carried out sinusoidally modulating sample temperature with amplitude of 0.5 K and a period of 80 s for at least 40 min. In the studied temperature range, an uncertainty of 3% was experimentally estimated for *C_p_* [26].

Density, *ρ*, was measured within the temperature range from 288.15 to 313.15 K by means of an oscillating U-tube densimeter DMA 500 (Anton Paar, Graz, Austria). Water and toluene were selected as reference materials to perform device calibration. Relative uncertainty of density measurements with this device was established to be lower than 0.1% [67].

Surface tension, *σ*, at the air–sample surface was studied by means of a DSA30 drop shape analyzer (Krüss GmBH, Hamburg, Germany). Tests were performed in a TC40 environmental chamber (also from Krüss GmBH), in which the sample temperature was stabilized from 288.15 to 328.15 K each 10 K. *σ* was obtained from the shape analysis of sample drops suspended at the apex of a vertical syringe (15-gauge needle with an outer diameter of 1.835 mm) based on the Young–Laplace equation. Reported results were calculated from the study of at least three different drops (with a minimum of 10 recordings each). Necessary density values were experimentally obtained in this work at 288.15, 298.15, and 308.15 K while predicted values, at 318.15 and 328.15 K, were obtained by a second-order polynomial fitting based on the temperature dependence of PEG400 densities measured in Marcos et al. [30] and the influence of adding PVP-capped Ag nanoparticles on this physical property determined in the present study, following a procedure similar to that used by Berrada et al. [68]. Experimental uncertainty of surface tension measurements was previously estimated to be better than 1% [24]. A more detailed description of the experimental device and followed procedure can be found in Gómez-Villarejo et al. [69].

## 3. Results and Discussion

### 3.1. Phase Change Characterization

**Figure 6:**
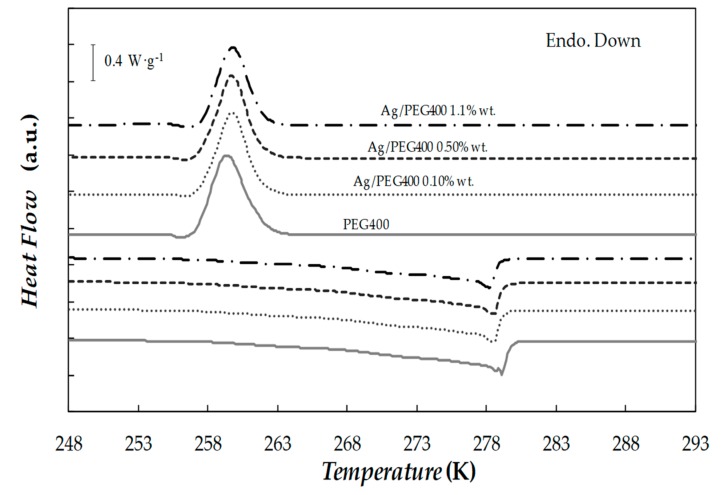
DSC cooling and heating thermograms obtained for base fluid and different Ag/PEG400 NePCMs at scanning rates of 2 K·min^−1^.

The solid–liquid phase transitions were studied by temperature scans at cooling/heating rates of 1, 2, 5, and 10 K·min^−1^ for PEG400 as base material, and three Ag/PEG400 mass concentrations (0.10%, 0.50%, 1.1%). After completing the necessary runs to study the samples at the predefined cooling/heating rates, some representative DSC scans were repeated to verify that no significant change occurred between the original test and replicate, and thus validate the characterization of phase change transitions. In addition, in order to analyze the reliability of designed materials, base PEG400 and NePCM loaded with 1.1% of silver nanoparticles were subjected to 100 heating–cooling cycles and no reduction in latent heat or shift in melting or solidification transitions was observed. As an example, Figure 6 shows the thermograms obtained at cooling and heating rates of 2 K·min^−1^.

As it can be observed, the addition of nanoparticles did not significantly modify the onset temperature of the freezing process. Thus, while recrystallization started at around ~259.3 K for pure PEG400, this transition occurred at ~259.8 K in the case of the NePCM loaded with the highest nanoparticle concentration. However, with increasing silver loading, a slight shift towards lower temperatures was found in the melting transition. These lower melting temperatures (due to the dispersion of nanoparticles), led to a reduction in sub-cooling of up to 7.1% in the case of the Ag(1.1 wt%)/PEG400 sample (in comparison with neat PEG400).

### 3.2. Isobaric Heat Capacity

**Figure 7:**
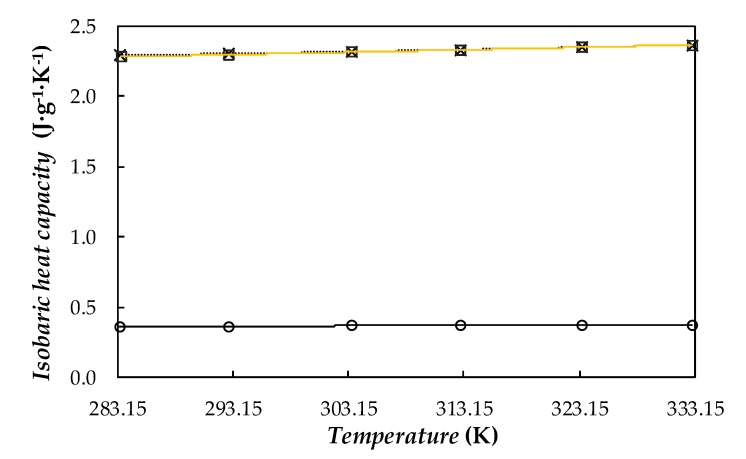
Temperature dependence of isobaric heat capacity of (×) PEG400, (○) dry Ag nanoparticles, and (□) Ag(0.50 wt%)/PEG400 nanofluid. (·····) Second-order polynomial fitting and (—) values provided for Ag(0.50 wt%)/PEG400 by using Equation (1).

Experimental isobaric heat capacities, *C_p_*, for PEG400, the dry powder of Ag nanoparticle, and the Ag(0.5 wt%)/PEG400 nanofluid in the temperature range from 283.15 to 333.15 K are shown in Figure 7.

Obtained values for base fluid exhibit a good agreement with data reported by Francesconi et al. [70] and Marcos et al. [30,32] for other poly(ethylene glycol) with similar average molecular weights, around 400 g·mol^−1^. Results measured for dry silver nanoparticles were also compared with the values recommended by Touloukian and Buyco [71] for bulk silver. In the case of the nanofluids, *C_p_* values were determined by using the following weighted-average equation [72,73]:

(1) $$c_{p,\mathrm{nf}}=\phi_{m,np}\cdot c_{p,\mathrm{np}}+\left(1-\phi_{m,np}\right)\cdot c_{p,\mathrm{bf}}$$

where $\phi_{m,np}$ is the mass fraction of nanoparticles, while np, bf, and nf subscripts stand for nanoparticles, base fluid, and nanofluid, respectively. For comparison, the values estimated for the sample prepared with the silver loading 0.5 wt% are also plotted in Figure 7. In the studied temperature range, the specific heat capacities experimentally measured for the silver nanopowder are ~84% lower than the values obtained for base PEG400 at the corresponding temperature. *C_p_* property slightly decreases with increasing mass concentration of silver nanoparticles, with reductions lower than 0.9% within studied concentration range of silver nanoparticles. This trend is in agreement with that predicted from *C_p_* data measured in our laboratory for the base fluid and dry PVP-capped Ag nanoparticles by using Equation (1). Other studies on nanofluids using PEG400 as base fluid found diminutions of 3% for the concentration of 1wt% of multiwalled carbon nanotubes (MWCNT) [32], or 0.34% for a dispersion of 0.5 wt% using functionalized graphene nanoplatelets [30]. Hence, it can be concluded that the addition of the PVP-capped Ag nanoparticles does not lead to a significant reduction in the sensible heat capacity of the phase change material.

### 3.3. Thermal Conductivity

**Figure 8:**
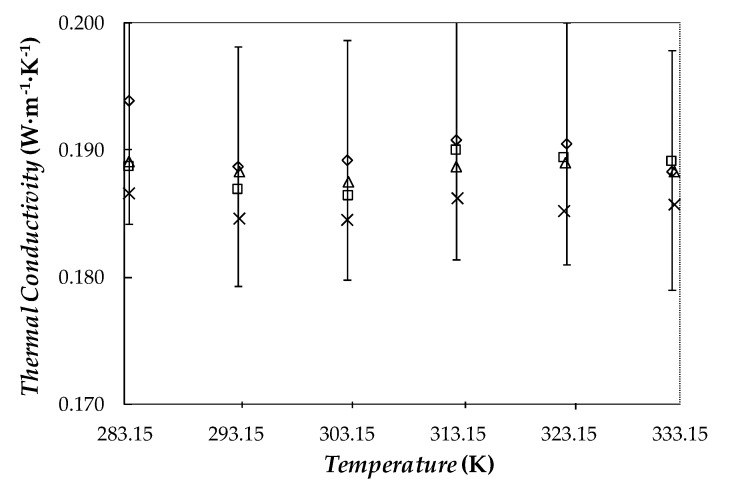
Temperature dependence of thermal conductivity for pure PEG400 (×) and the Ag/PEG400 nanofluids: (△) 0.10 wt%, (□) 0.50 wt% and (◇) 1.1 wt%. Error bars indicate 5% uncertainty regarding experimental data.

Experimental thermal conductivities obtained for the base fluid and the three Ag/PEG400 dispersions are shown in Figure 8. As it can be observed, the addition of nanoparticles slightly improved the thermal conductivity of the base phase change material. Those enhancements rose with increasing nanoparticle loading, a maximum improvement of 3.9% being reached in the case of the 1.1 wt% concentration. Other studies conducted with PEG400 as base fluid reported higher increases in thermal conductivity, 12.7% for 1 wt% MWCNT/PEG400 nanofluid [32] and 23% for a PEG400 dispersion containing 0.5 wt% of functionalized graphene nanoplatelets (fGnP) [30]. The PVP-capped procedure carried out with silver nanoparticles to favor the stability of the conceived dispersions entailed a penalty in the expected improvement of the intrinsic heat transfer of the sample. According to the results shown in the Figure 8, a slight improvement in thermal conductivity was observed. Modifications in this property were within the experimental uncertainty of this device. In any case, an upward trend with increasing mass fraction was obtained.

Over the last century, huge research efforts have been directed towards understanding and theoretically describing the thermal conductivity of solid–liquid colloidal systems.

In this work, results experimentally measured for Ag/PEG400 NePCMs were compared with the values provided by using some representative theoretical or semi-empirical equations. Maxwell [74] proposed the first equation to estimate the thermal conductivity of solid–liquid suspensions (in our case nanofluids, *k*_nf_) from the volume concentration of particles, ($\phi_{v,np}$), and the thermal conductivities of base fluid, *k*_bf_, and particles, *k*_np_:

(2) $$k_{\mathrm{nf}}=\frac{k_{\mathrm{np}}{+2k}_{\mathrm{bf}}+{2(k}_{\mathrm{np}}-k_{\mathrm{bf}})\phi_{v,np}}{k_{\mathrm{np}}{+2k}_{\mathrm{bf}}-{(k}_{\mathrm{np}}-k_{\mathrm{bf}})\phi_{v,np}}k_{\mathrm{bf}}$$

However, the Maxwell model does not take into account several parameters such as particle size, agglomeration, or temperature, while those parameters have been found to strongly influence the thermal conductivity of several nanofluid systems [75,76,77]. Different studies have been developed [75,76,77,78,79] in order to identify the main mechanisms governing thermal conductivity in the solid–liquid interface.

Murshed et al. [80] proposed a model that considers the size of dispersed particles but also suggests the existence of an interfacial layer in which thermal conductivity takes an intermediate value between those of the base fluid and nanoparticles. According to the fundamental theory behind interfacial thermal resistance, heat exchange through the solid–liquid interfacial layer is an important function of the affinity between the two phases [81]. The Murshed et al. [80] equation can be expressed for spherical nanoparticles as follows:

(3) $$k_{\mathrm{nf}}=\frac{\left(k_{\mathrm{np}}-k_{\mathrm{lr}}\right)\phi_{v,np}k_{\mathrm{lr}}\left(2\gamma_{1}^{3}-\gamma^{3}+1\right)+\left(k_{\mathrm{np}}{+2k}_{\mathrm{lr}}\right)\gamma_{1}^{3}\left(\left(\phi_{v,np}\gamma^{3}\left(k_{\mathrm{lr}}-k_{\mathrm{bf}}\right){+k}_{\mathrm{bf}}\right)\right)}{\gamma_{1}^{3}\left(k_{\mathrm{np}}{+2k}_{\mathrm{lr}}\right)-\left(k_{\mathrm{np}}-k_{\mathrm{lr}}\right)\phi_{v,np}\left(\gamma_{1}^{3}{+\gamma }^{3}-1\right)}$$

where $\phi_{v,np}$ is the volume fraction of the nanoparticles in suspension, *γ_1_* = 1 + *h*/*r* and *γ* = 1 + *h*/2·*r* relationships depend on the thickness of interfacial layer (*h*) and radius (*r*); while *k*_lr_, *k*_np_, and *k*_bf_ are the thermal conductivities of the interfacial layer, nanoparticles, and base fluid, respectively. In our analysis, a value of *h* = 12.5 nm was assumed for that interfacial layer considering that nanoparticles are spherical and the STEM and DLS average sizes are ~22 nm and ~53 nm (measurement of day 0), respectively.

**Figure 9:**
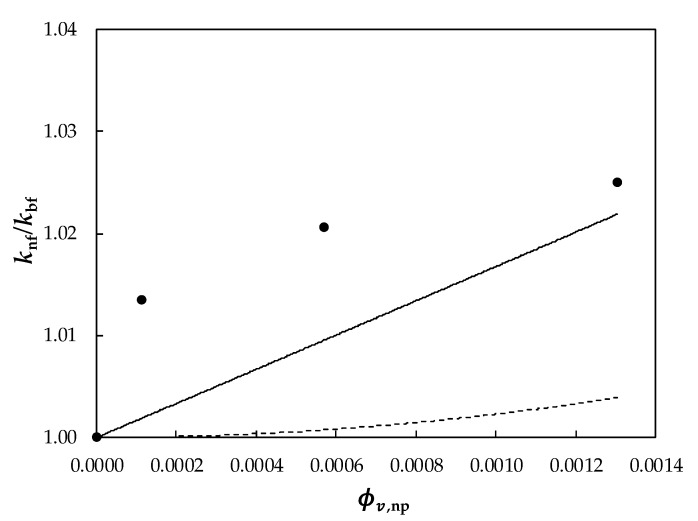
Relative thermal conductivity, *k*_nf_/*k*_bf_, as a function of volume fraction, $\phi_{v,np}$, for Ag/PEG400 nanofluids at 313.15 K. (●) Experimental results obtained in this work and predicted values by (- - -) Maxwell [74] and (—) Murshed et al. [80] models.

A comparison between the experimental relative thermal conductivities and the values predicted by using the Maxwell and Murshed equations is graphically presented in Figure 9.

A thermal conductivity of *k*_np_ = 429 W·m^−1^·K^−1^ was considered in this work for the silver nanoparticles [71]. As it can be observed, experimental thermal conductivities for the nanofluids are larger than the values predicted by the Maxwell and Murshed models. For the highest nanoparticle loading, 1.1 wt% of silver, absolute average differences, *AAD%*, between experimental and theoretical data sets reach 2.1% (Maxwell) and 0.5% (Murshed). Other studies conducted with PEG400 as base fluid reported that Hamilton–Crosser, Murshed, Xue, or Nan models also underestimated thermal conductivity results. So, differences of 5.6% (H–C), 5.5% (Murshed) and 2.6% (Xue), were obtained when dispersed MWCNT [32] while the Nan correlation allowed obtaining 1% for GnP suspensions [30]. The cause of those larger experimental enhancements in thermal conductivity may be due to the size dependence of thermal conductivity. The smaller the particle size, the larger is the surface–volume ratio and, consequently, the heat transfer capacity of the nanoparticles also increases [82,83]. However, in the case of metallic nanoparticles, this is not always true. As an example, reductions in the thermal conductivity of copper or silver nanoparticles with decreasing particle size were previously published in the literature by Warrier and Teja [84] or Nath and Chopra [85].

### 3.4. Density

**Figure 10:**
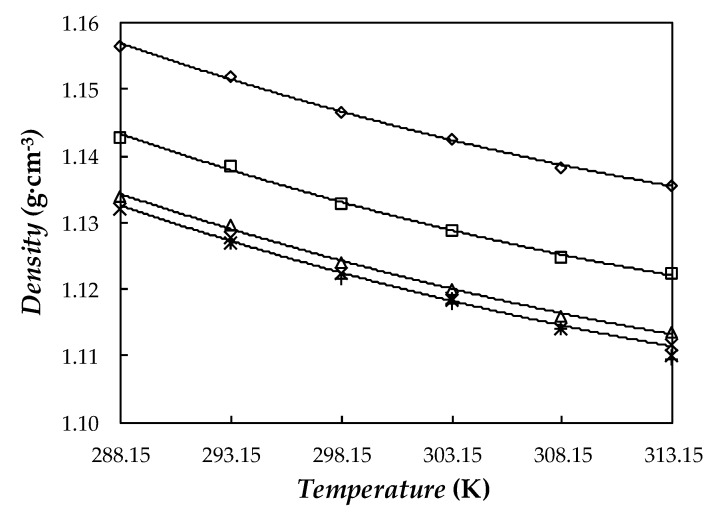
Temperature dependence of density (*ρ*) for: (×) base PEG400, (△) 0.10 wt%, (□) 0.50 wt%, and (◇) 1.1 wt% NePCMs. (—) Second-order polynomial fittings. Values reported for (+) PEG400 by Han et al. [88] and for (*) PEG600 by Trivedi, Bhanot and Pandey [86].

Densities of base fluid and NePCMs at silver concentrations of 0.10, 0.50, and 1.1 wt% were measured at atmospheric pressure in the temperature range from 288.15 to 313.15 K. Experimental results are displayed for the four samples in Figure 10. According to the normal PEG density dependence on polymer molar mass [86], results obtained in this work for base PEG400 agree well with the *ρ* values reported in the literature for other poly(ethylene glycol) with similar molecular mass [58,86,87,88]. With the objective of evidencing this good agreement, some of those literature results are also plotted in Figure 10. As an example, *ρ* value determined in this study for PEG400 (*M_n_* ≈ 520 g·mol^−1^) at 298.15 K is 0.07% higher than the result reported for PEG400 (*M_n_* = 415 g·mol^−1^) by [88] and 0.06% lower than the data provided for PEG600 by Trivedi, Bhanot and Pandey [86] at 293.15 K.

Density rises with the addition of nanoparticles and these enhancements do not depend on temperature. Average *ρ* rises (regarding base PEG400) for 0.10, 0.50, and 1.1 wt% PVP-capped Ag loadings are 0.16%, 0.94%, and 2.2%, respectively. Other studies in which PEG400 was used as base fluid observed a maximum increase of 0.42% for 1 wt% MWCNT/PEG400 nanofluid [32] or 0.33% for 0.5 wt% GnP/PEG400 one [30]. Our density modifications (2.2%) for the NePCM containing 0.10 wt% of PVP-capped silver nanoparticles are slightly larger than 0.03% reported by Nakhjavani et al. [89] for a 0.1 wt% Ag/water nanofluid or 0.09% to 0.11% reported by Bahiraei and Heshmatian [90] and Yarmand et al. [91] for aqueous hybrid nanofluids containing 0.1 wt% of graphene-decorated silver nanoadditives. Experimental results here obtained for Ag/PEG400 dispersions were also compared with the values provided by using the following weight-average equation:

(4) $$\frac{1}{\rho_{n_{f}}}=\frac{\phi_{m,\;np}}{\rho_{np}}+\frac{\phi_{m,\;sf}}{\rho_{sf}}+\frac{1-\phi_{m,\;np}-\phi_{m,\;sf}}{\rho_{bf}}$$

where $\phi_{m,np}$ and $\phi_{m,sf}$ are the nanoparticle and surfactant volume fractions, while nf, np, sf, and bf subscripts stand for nanofluid, nanoparticle, surfactant, and base fluid, respectively. Maximum deviations between experimental results and values provided by Equation (5) are 1.1%.

As expected, density decreases with increasing temperature. This temperature dependence can be fitted with *AADs%* of 0.02% using second-order polynomial fittings. In the studied range, average density modifications each 5 K are 0.38% for base PEG400 and 1.1 wt% Ag/PEG400 considering a value of *ρ*_np_ = 10.49 g·cm^−3^ corresponding to crystalline silver [52].

### 3.5. Thermal Diffusivity

The higher the thermal diffusivity, *α*, of a material is, the faster the thermal energy is propagated. For that reason, thermal diffusivity becomes even more important than thermal conductivity when selecting NePCMs for energy storage. *α* is related to thermal conductivity, *k*, and heat capacity per unit of volume, *ρ·C_p_*, throughout the following expression:

(5) $$\alpha =\frac{k}{\rho \cdot c_{p}}$$

Thermal diffusivities were calculated for base PEG400 and designed NePCMs from *k*, *ρ*, and *C_p_* data above presented for these materials. In the studied temperature range, neat PEG400 exhibits *α* values from 7.13·10^−8^ to 7.15·10^−8^ m^2^/s. These results are similar to the data reported in literature for other poly(ethylene glycol) with similar molecular mass [30]. Maximum improvements in thermal diffusivity were obtained for the highest concentration (1.1 wt%) nanoparticle loading, for which increases lower than 2% were obtained.

### 3.6. Viscosity

**Figure 11:**
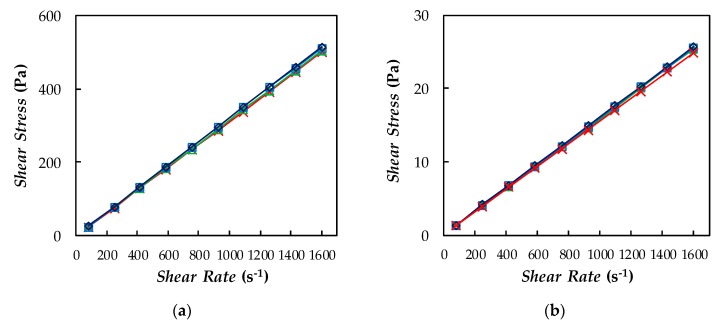

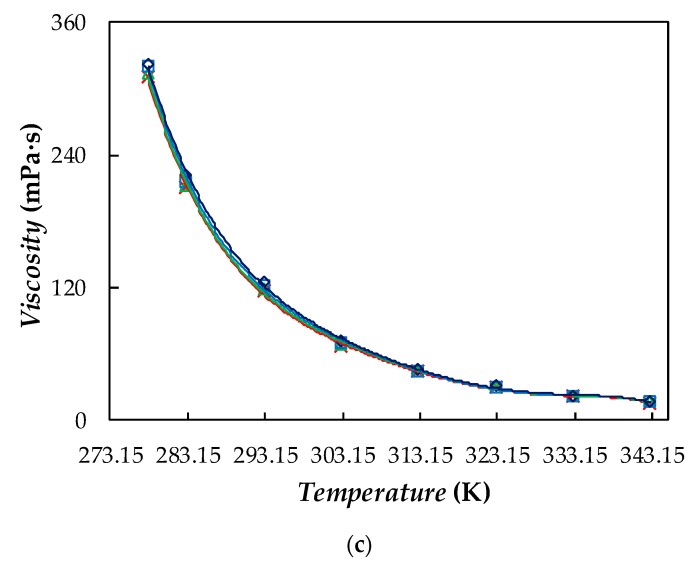
(**a**,**b**) Relationship between shear stress and shear rate at (**a**) 278 K and (**b**) 343 K. (**c**) Temperature dependence of dynamic viscosity. (×) base PEG400, 0 wt%; (△) 0.1 wt%; (□) 0.5 wt%; and (◇) 1.1 wt% Ag/PEG400 NePCMs. (—) Linear fittings, in (**a**,**b**); or VTF equation, Equation (6), in (**c**).

Shear viscosity was studied for base PEG400 and the three formulated NePCMs in the temperature range between 278.15 and 343.15 K. Taking into account that the dynamic viscosity of poly(ethylene glycol) is expected to increase with polymeric molecular mass [92], a good agreement is observed when comparing *η* values here obtained for base PEG400 (*M_n_* = 520 g·mol^−1^) and results previously reported for other PEGs [30,32,58,88]. As an example, Figure 11a,b shows shear rate–shear stress flow curves obtained for the four samples at 278.15 and 343.15 K.

A linear rise in shear stress with increasing shear rate was observed for all studied samples. This linear relationship, which is temperature independent, confirmed that like base fluid, designed dispersions are Newtonian. As pointed out by [93], this fact can be interpreted as an indication of the quality of dispersions and lack of agglomerates or aggregates in proposed NePCMs. This result agrees with the Newtonian behavior in the shear rate region of 1–200 s^−1^ observed by Tamjid and Guenther [94] when investigating di(ethylene glycol)-based nanofluids containing 0.11% and 0.22% volume concentrations of silver nanoparticles. However, as reported in that study, larger amounts of nanoparticles led to a non-Newtonian or pseudoplastic behavior.

The evolution of dynamic viscosity with temperature is depicted in Figure 11c. As expected, this property exponentially decreased with increasing temperature. This behavior can be described by using the well-known Vogel–Fulcher–Tammann (VFT) equation:

(6) $${\;\ln\eta =\ln\eta }_{0}+\frac{{D\cdot T}_{0}}{{T-T}_{0}}$$

where *η*_0_, *D,* and *T*_0_ are the fitting parameters. Table 1 reports the values of those three adjustable coefficients as well as *AADs%* between our experimental results and those values fitted by the VFT model.

The good description (with *AADs%* better than 2.2%) of the Vogel–Fulcher–Tammann equation is shown in Figure 11c. *D* coefficient is also known as the Angell strength parameter, while its inverse, *F* = 1/*D*, was defined as fragility by Angell et al. [95]. Studied NePCMs exhibited Angell strength coefficients similar to those of poly(propylene glycol) dimethyl ether [96] or ethylene glycol [97]. Reduced values of the *D* parameter were an indication of fluid fragility and that liquid configurational structure rapidly breaks down with rising temperature [98].

**Figure 12:**
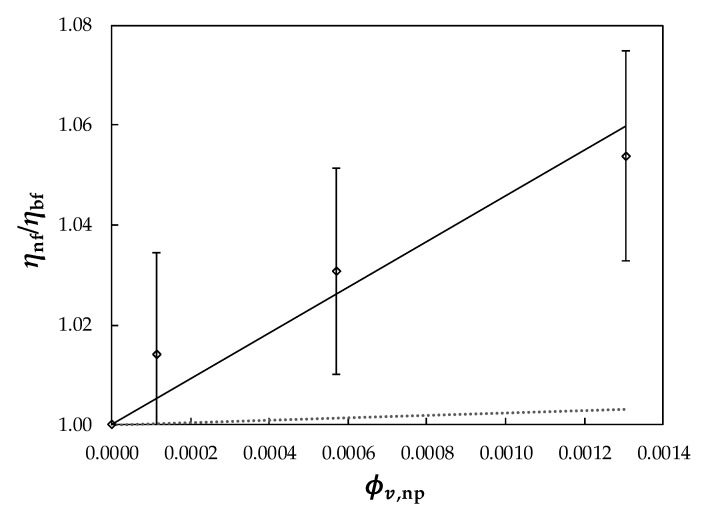
Viscosity ratios *η*_nf_/*η*_bf_ vs. silver nanoparticles volume fraction, ($\phi_{v}$, _np_) at 313.15 K. (◇), experimental values; (·····) Einstein; (—) Equations (7) and (8). Error bars indicate 2% uncertainty.

On the other hand, dynamic viscosity increased with nanoparticle loading. In Figure 12, the dynamic viscosity dependence with volume fraction is represented. In this case, average viscosity rises were 1.4%, 2.8%, and 5.4% for Ag/PEG400 dispersions containing 0.011%, 0.057%, and 0.13% in volume fraction of PVP-capped silver nanoparticles, respectively. These modifications were significantly lower than those reported by Zadeh and Toghraie [99] in their study on Ag–EG nanofluids, in which nanofluid apparent dynamic viscosity at 318 K rose by more than 90% as volume fraction increased from 0.25% to 2%.

Different equations have been proposed in the literature to describe *η*($\phi_{v}$) dependence of solid–liquid suspensions [22]. For dilute non-interacting suspensions of spherical-shaped particles, the well-known Einstein [100] predicted that viscosity linearly increases as a function of volume concentration:

(7) $$\eta_{r}=1+2.5\;\phi_{v,np}$$

where *η_r_* = *η*/*η_0_* is the so-called reduced viscosity and $\phi_{v,np}$ is the nanoparticle volume concentration. With rising nanoparticle concentration, nanofluid viscosity usually increases in a non-linear manner and, consequently, the Einstein relationship may greatly underpredict *η* data.

According to Chow [101], the *η*($\phi_{v,np}$) relationship of Newtonian colloids can be formally written as a virial of series:

(8) $$\eta_{r}=\frac{\eta }{\eta_{0}}=1+\overset{N}{\underset{i=1}{\sum }}c_{i}\cdot \phi_{v,np}^{i}$$

where *N* is the degree of expansion and *c_i_* are the fitting parameters which may vary from one sample to another [102].

Figure 12 shows a graphical comparison between experimental relative viscosities and the values provided by the predictive Einstein (1906) equation or a linear (*N* = 1) correlation based on Equation (8). In this case, values predicted using Einstein (1906) show *AADs*% with experimental data of 3%. A better description with an *AAD%* of 0.22%, was obtained utilizing a linear fitting based on Equation (8) with *c*_1_ = 43.

### 3.7. Surface Tension

**Figure 13:**
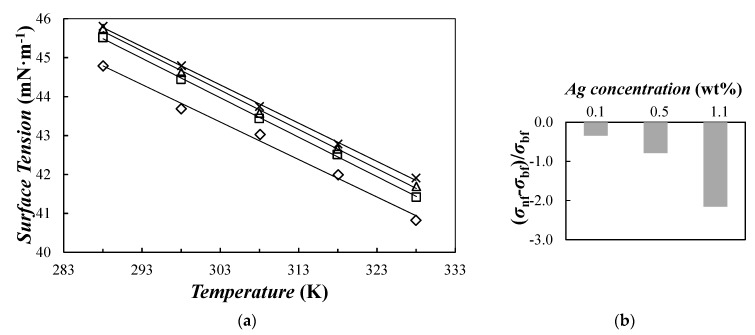
(**a**) Temperature dependence of surface tension: (×) base fluid, (△) 0.10 wt%, (□) 0.50 wt%, and (◇) 1.1 wt% nanoparticle concentrations of Ag/PEG400 nanofluids. (—) First–order polynomial fittings. (**b**) Average modifications in surface tension regarding base fluid, i.e., (*σ*_nf_–*σ*_bf_)/*σ*_bf_.

Surface tension at the air–sample surface was determined for base PEG400 and Ag/PEG400 suspensions. Obtained results for the four samples are depicted in Figure 13.

Our experimental values for base fluid showed maximum deviations lower than 0.9% with previous results reported in the range from 298 to 318 K for a similar PEG by Fu et al. [103]. Surface tension decreased with increasing temperature. In the studied temperature range, reductions of ~0.21–0.22% each 10 K were observed for base fluid and formulated NePCMs. This downward trend with increasing temperature can be described by using a first-order polynomial fitting with *ADDs%* lower than 0.25%. Lower surface tensions were also measured for formulated NePCMs when compared with neat PEG400 (see Figure 13b). These diminutions, that reach 2.2% for the Ag(1.1 wt%)/PEG400 sample, can be attributed to the presence of PVP surfactant used to stabilize silver nanoparticles (PVP:Ag ratio of 0.068) or other reagents remaining in the parent NePCM from the formulation process. Surfactants are surface active molecules that improve suspension stability by recovering nanoparticles and modifying particle–surface interaction forces. Surfactant molecules also place at the air–sample surface, which in turn, reduces nanofluid surface tension.

## 4. Conclusions

Three dispersions of PVP-capped silver nanoparticles in a poly(ethylene glycol) PEG400 at nanoparticle mass concentrations from 0.10% to 1.1% were specifically synthesized for this study. Such nanofluids were characterized for the purpose of being used as potential nano-enhanced phase change materials. The poly(ethylene glycol) utilized as base fluid was an almost monodisperse polymer with an average mass molar mass of 533 g·mol^−1^. Dry silver nanoparticles exhibited a quasi-spherical morphology with an average diameter of ~22 nm. Once suspended in PEG400, nanoparticles showed DLS hydrodynamic diameters of ~50 nm, a value that remained constant over time indicating that no significant sedimentation or agglomeration occurred in the dispersion. The dispersion of PVP-capped silver nanoparticles improved the thermal conductivity of nanofluids, maximum enhancements reaching 3.9% for the Ag(1.1 wt%)/PEG400 sample. Calorimetry analyses showed that the addition of silver nanoparticles slightly reduced undesirable sub-cooling phenomena, in which a maximum improvement of 7.1% was found for the highest nanoparticle loading. Also at 1.1 wt% silver content, modifications in isobaric heat capacity, density, and surface tension were 0.9%, 2.2% and 2.2%, respectively. Studied NePCMs showed a Newtonian behavior with average increases ranging from 1.4% to 5.4%, in comparison to neat poly(ethylene glycol). Obtained improvements in the sub-cooling phenomenon and thermal conductivity evidence the potential that nanoparticle addition has in the development of phase change materials with enhanced thermal properties. However, more research in the selection of nanoadditives and the design of NePCMs is still necessary so that those materials achieve a competitive edge over conventional thermal storage materials.

## Figures and Tables

**Table 1:** *η*_0_, *D,* and *T*_0_ fitting parameters, standard deviations, *s*, and *AADs%.* from the VFT equation, Equation (6), at different mass fractions.

	Base Fluid (0 wt%)	0.10 wt%	0.50 wt%	1.1 wt%
*η*_0_/mPa·s	0.0659	0.0672	0.0239	0.0246
*D*	6.37	6.37	9.93	9.92
*T*_0_/K	158.19	158.08	135.41	135.57
*s*/mPa·s	1.4	1.3	2.4	2.1
*AAD%*	1.2%	1.1%	2.2%	2.0%
